# Genome-wide profiling reveals alternative polyadenylation of mRNA in human non-small cell lung cancer

**DOI:** 10.1186/s12967-019-1986-0

**Published:** 2019-08-07

**Authors:** Shirong Zhang, Xiaochen Zhang, Wei Lei, Jiafeng Liang, Yasi Xu, Hailiang Liu, Shenglin Ma

**Affiliations:** 1 Department of Translation Medicine Centre, Affiliated Hangzhou First People’s Hospital, Zhejiang University School of Medicine, Hangzhou, 310006 People’s Republic of China; 2 Department of Oncology, Affiliated Hangzhou First People’s Hospital, Zhejiang University School of Medicine, No. 261 Huansha Road, Shangchen District, Hangzhou, 310006 People’s Republic of China; 3 Department of Medical Oncology, The First Affiliated Hospital, Zhejiang University School of Medicine, Hangzhou, China; 4 CapitalBio Genomics Co., Ltd, Dongguan, 523808 People’s Republic of China; 5 CapitalBio Technology Inc, Beijing, 101111 People’s Republic of China

**Keywords:** Lung cancer, 3′UTR, Poly(A) processing, CSTF2

## Abstract

**Background:**

Lung cancer is the second most common cancer with an extremely poor overall survival rate. Post-transcriptional regulation of gene expression play many important roles in human cancer, and one of the potential mechanisms underlying this is alternative mRNA maturation at its 3′ untranslated regions (3′-UTRs).

**Methods:**

Cancer tissues and paired adjacent normal lung tissues from 26 patients diagnosed with non-small cell lung cancer (NSCLC) were analyzed by in vitro transcription-sequencing alternative polyadenylation sites (IVT-SAPAS). 41,773,101 reads in average were obtained from each paired sample. A potential regulation of Cleavage Stimulation Factor Subunit 2 (CSTF2) on 3′UTR length of genes was tested in H460 cells.

**Results:**

1439 (10.26%) genes showed up-regulated expression and 1364 (9.72%) genes showed down-regulated expression in lung cancer tissue versus normal lung tissue, and shorten 3′UTR in cancer tissue was detected in cancer tissues collected from 96.2% (25/26) patients, indicating lung cancer tend to have shortened 3′UTRs of these identified genes. KEGG analysis showed 1855 genes with shorten 3′UTR were enriched in mTOR signaling, ubiquitin mediated proteolysis and RNA degradation. Knocking down CSTF2 expression in H460 cells results in 3′UTR elongation of genes that was identified to be with shortened length in cancer tissues.

**Conclusion:**

Alternative polyadenylation (APA) site-switching of 3′UTRs is prevalent in NSCLC, and CSTF2 may serve as an oncogene regulates the 3′UTR length of cancer related genes in NSCLC.

**Electronic supplementary material:**

The online version of this article (10.1186/s12967-019-1986-0) contains supplementary material, which is available to authorized users.

## Introduction

Lung cancer is the second most common cancer and the leading cause of cancer-related death for both man and woman worldwide, with an extremely poor overall survival rate, 5-year survival rate is only approximate 18% [1]. Although, lung cancer can be divided into many subtypes, historically lung cancer can be classified into two distinct types according to clinical tumor behaviors, small cell lung carcinoma (SCLC) and non–small cell lung carcinoma (NSCLC) [2]. NSCLC is the most common type of lung cancer, accounting for 80–85% of all lung cancer cases, and, as a major subtype, about 45% to 50% of NSCLC is diagnosed as lung adenocarcinoma, 30–35% is squamous cell carcinomas [3, 4].

Cancer is a disease with multiple genetic alternations. For example, proto-oncogenes are often activated in cancer cells and contribute to the tumorigenesis. The activation of oncogene can be caused by a mutation that leads to constitutive activation of the encoded proteins or by a mutation that induce gene expression, the latter occur through mechanisms like amplification of the gene copy number or chromosomal translocations resulting in replacement with a constitutive active promoter [5]. However, studies have revealed that, in addition to these genetic alterations, post-transcriptional regulation of gene expression also play many important roles in cancer development and progression, and one of the potential mechanisms underlying this is alternative mRNA maturation at its 3′ untranslated regions (3′-UTRs) which leads to distinct mRNA isoforms from the same gene [6, 7].

The architecture of pre-mRNA consists of coding regions and non-coding regions located separately at the 5′ and 3′ end of mRNA. The 3′ ends of almost all eukaryotic mRNAs are formed in a two-step process, an endonucleolytic cleavage followed by polyadenylation (the addition of a poly-adenosine or poly(A) tail) [8]. The formation of 3′-UTRs through polyadenylation process of sequential addition of a poly(A) tail on 3′ end of precursor mRNA is the last key step in mRNA maturation process [9].

3′-UTR of mRNA often contains many binding sites for regulatory RNA-binding proteins and microRNAs, and thus the length of 3′-UTR is important for regulation of mRNA stability, localization and protein translation efficiency [10]. Alternative polyadenylation (APA), a phenomenon that RNA molecules with different 3′ ends originate from distinct polyadenylation sites of a single gene, can often results in mRNA isoforms with the same coding sequence but different lengths of 3′-UTRs, which is the last step of the post-transcriptional processing. APA has been discovered to be an emerging mechanism of regulating gene expression [11]. Of interest, cancer cells, or similarly proliferating cells like early-stage embryonic cells, often express substantial amounts of mRNA isoforms with shorter 3′-UTRs [6, 12]. Shortening of 3′-UTRs has also been found to correlate with poor prognosis of cancer patients, including lung cancer [13, 14]. However, the detailed mechanism of how APA is regulated in normal and in cancer cells remains to be elucidated.

In this study, we used paired clinical tissue specimen to determine the differential expressed genes and the tandem 3′-UTR lengths of these genes in NSCLC. We also examined the potential protein factors that may influence the observed change of the 3′-UTR length. Our results showed that shorten 3′-UTRs is a common feature for the genes that are differentially expressed in cancer cells, and expressions of 3′ end processing protein factors cleavage stimulation factor (CSTF) may play vital role for the shortening of 3′-UTR.

## Materials and methods

### Patient information and tissue specimens

Twenty-six patients diagnosed with NSCLC (Table 1) were enrolled in this study. The protocol for using paired cancerous tissues and adjacent normal lung tissues was approved by Ethics Committee of The First People’s Hospital of Hangzhou. Fresh tissues were collected after surgery from each patient. All samples were snap frozen in liquid nitrogen within 30 min after surgical removal and then stored at − 80 °C. H&E staining was performed for validation of pathologic status of each specimen.

**Table 1:** The clinical information about 26 patient diagnosed with NSCLC

No.	Sex	Age (years)	Smoking history (per month)	Pathologic type	TNM classification	Clinical stage
1	Women	73	No	Adenocarcinoma	T2aN1M0	IIB
2	Men	76	Yes	Adenocarcinoma	T2aN2M0	IIIA
3	Men	66	Yes	Adenocarcinoma	T2aN0M0	IB
4	Men	67	No	Squamous	T1cN0M0	IA
5	Women	62	No	Adenocarcinoma	T2aN1M0	IIB
6	Women	72	No	Adenocarcinoma	T2aN1M0	IIB
7	Women	78	No	Squamous	T2aN2M0	IIIA
8	Women	67	No	Squamous	T2aN0M0	IB
9	Man	49	Yes	Squamous	T4N0M0	IIIA
10	Women	63	No	Adenocarcinoma	T4N1M0	IIIA
11	Man	81	Yes	Adenocarcinoma	T2aN2M0	IIIA
12	Man	60	Yes	Adenocarcinoma	T2N2M0	IIIA
13	Man	59	Yes	Adenocarcinoma	T2aN0M0	IB
14	Women	49	No	Adenocarcinoma	T2N2M0	IIIA
15	Women	74	No	Adenocarcinoma	T2aN0M0	IB
16	Man	55	Yes	Adenocarcinoma	T1cN0M0	IA
17	Women	64	No	Adenocarcinoma	T2aN2M0	IIIA
18	Man	79	No	Adenocarcinoma	T1bN0M0	IA
19	Man	69	Yes	Squamous	T3N1M0	IIIA
20	Man	49	Yes	Squamous	T1bN0M0	IA
21	Man	63	No	Adenocarcinoma	T1cN1M0	IIB
22	Man	54	Yes	Adenocarcinoma	T2N2M0	IIIA
23	Women	71	No	Adenocarcinoma	T2aN1M0	IIB
24	Man	47	No	Adenocarcinoma	T1bN0M0	IA
25	Man	61	Yes	Squamous	T1cN2M0	IIIA
26	Women	58	No	Squamous	T2aN0M0	IB

### Cells culture

Human fetal lung fibroblast cell line HFL1 and lung cancer cell lines was purchased from ATCC (https://www.atcc.org/) and cultured in DMEM medium (Gibco, Waltham, MA, USA) supplemented with 10% fetal bovine serum (Gibco, Waltham, Massachusetts, USA), 100 U/mL penicillin and 100 μg/mL streptomycin at 37 °C humidified atmosphere with 5% CO_2_. Log-phase growing cells were collected for subsequent gene expression detection and RNA interference experiment.

### RNA interference

oligo siRNA (GGTGGATCGTTCTCTACGT) designed to target CSTF2 (Cleavage Stimulation Factor Subunit 2) was synthesized by RiboBio Co., Ltd. (Guangzhou, China). CSTF2 oligo siRNA was transfected into H460 cells at a concentration of 50 nM using Lipofectamine RNAiMAX Reagent (Invitrogen, Carlsbad, CA, USA). Cells were collected 48 h later after transfection for subsequent experiments, and quantitative real-time PCR was used to validate the efficiency of the RNA interference. Scramble siRNA Oligo was used as a control.

### Total RNA extraction and in vitro transcription-sequencing alternative polyadenylation sites (IVT-SAPAS) analysis

Total RNA were extracted from lung cancerous and paired adjacent normal tissues, or from cultured H460 cells using TRIzol reagent (Life Technologies, USA) according to the manufacturer’s protocol. The RNA samples were used to generate libraries for IVT-SAPAS analysis. The IVT-SAPAS was performed with the illumina HiSeq 2500 platform according to the manufacturer’s protocol [15]. Briefly, twelve libraries with distinct barcodes were pooled together and were sequenced with Hiseq 2500 with rapid run mode, and 20 dark cycles were taken and then 55 bp were further sequenced.

### Analysis of APA sites

The raw reads from IVT-SAPAS analysis were mapped to the human genome (hg19) using Bowtie software [16], and internal priming was filtered by our in-house Perl scripts. Poly(A) sites of each sample were defined by clustering the unique mapped reads, and were then merged together across samples. The merged poly(A) sites were mapped to the 3′UTRs dataset constructed from UCSC known genes for further analysis. The expression levels of poly(A) sites were calculated by the clustered reads and scaled to the sample with the lowest number of raw reads.

To compare the 3′-UTR length across samples, we standardized the length by defining the longest 3′UTR as 1.0 and calculated the weighted mean of 3′-UTR length according to the reads and related position of multiple poly(A) sites for each gene. Genes with alternative poly(A) were identified by a test of linear trend with significant P-values paired to a false discovery rate with cutoff of 1% between the lung cancer tissue and paired para-carcinoma tissue. We denoted the 3′-UTR length of each gene identified in paired non-carcinoma tissue as 1 and that in cancerous tissue as 2, and used Pearson correlation to test the relationship between two variables. A positive *R*-value indicates that the genes in the cancerous tissue showing longer tandem 3′UTRs versus the paired non-cancerous tissue, and a negative *R*-value indicates that the genes in the cancerous tissue showing shorter tandem 3′UTRs versus the paired non-cancerous tissue.

Functional annotation of the DEGs and APA switching genes was performed using the DAVID (The Database for Annotation, Visualization and Integrated Discovery) Bioinformatics Resources (http://david.abcc.ncifcrf.gov/), and genes with detected 3′UTRs length change were categorized for their significant enrichment according to Biological Process GO terms and pathways, with normalization of the background model for all transcripts found in both lung cancer and para-carcinoma tissues.

### Quantitative real-time PCR

Total RNA extracted from cells using TRIzol Reagent (Life Technologies, USA) was used for cDNA synthesis with using SuperScript^®^ III Reverse Transcriptase (Invitrogen, Carlsbad, CA, USA) and an oligo (dT) primer. 1.0 μg of total RNA was used for reverse transcription (RT) according to the manufacturer’s recommendations. qRT-PCR was performed in 10 μL of quantitative reaction mixtures containing 5 μL of SYBR Premix ExTaq (Takara, Japan), 0.4 μM each of forward and reverse primers, 1 μL cDNA, and nuclease-free water. The program used for qRT-PCR consisted of a denaturing cycle of 2 min at 95 °C, 45 cycles of PCR (95 °C for 10 s, 60 °C for 30 s) and a melting cycle of 60 °C for 15 s. The primer sequences is listed as below: CSTF2 qF 5′-TCGTTCTCTACGTTCTGTGTTCGTG-3′; CSTF2 qR 5′-ACCCTTTGGCTTTCCTGTCTCTCT-3′; β-actin 5′-qFGAGAAAATCTGGCACCACACCTT-3′; β-actin qR 5′-GCACAGCCTGGATAGCAACGTA-3′; The mRNA level of CSTF2 gene in H460 cells was normalized to β-actin mRNA level. Results of real-time PCR were analyzed using the 2^−ΔCT^ method to compare the transcriptional levels of CSTF2 gene in lung cancer cells to that in normal control.

### Western blotting analysis

Cells were collected and lysed in RIPA buffer containing protease and phosphatase inhibitors. 20 μg of cell lysates were used for western blot analyses using antibodies against CSTF2 and GAPDH (Cell Signaling Technology, USA). Simply, proteins were separated by SDS-PAGE and transferred to the polyvinylidene difluoride membrane. The membrane was then incubated with primary antibody at 4 °C overnight, followed by incubation of secondary antibody for one hour. Finally, the target proteins detected by chemiluminescence kit. The western blot signal was detected by ChemiDoc XRS + (BioRad, USA).

## Results

### Summary statistics of the IVT-SAPAS data in clinical samples

**Fig. 1:**
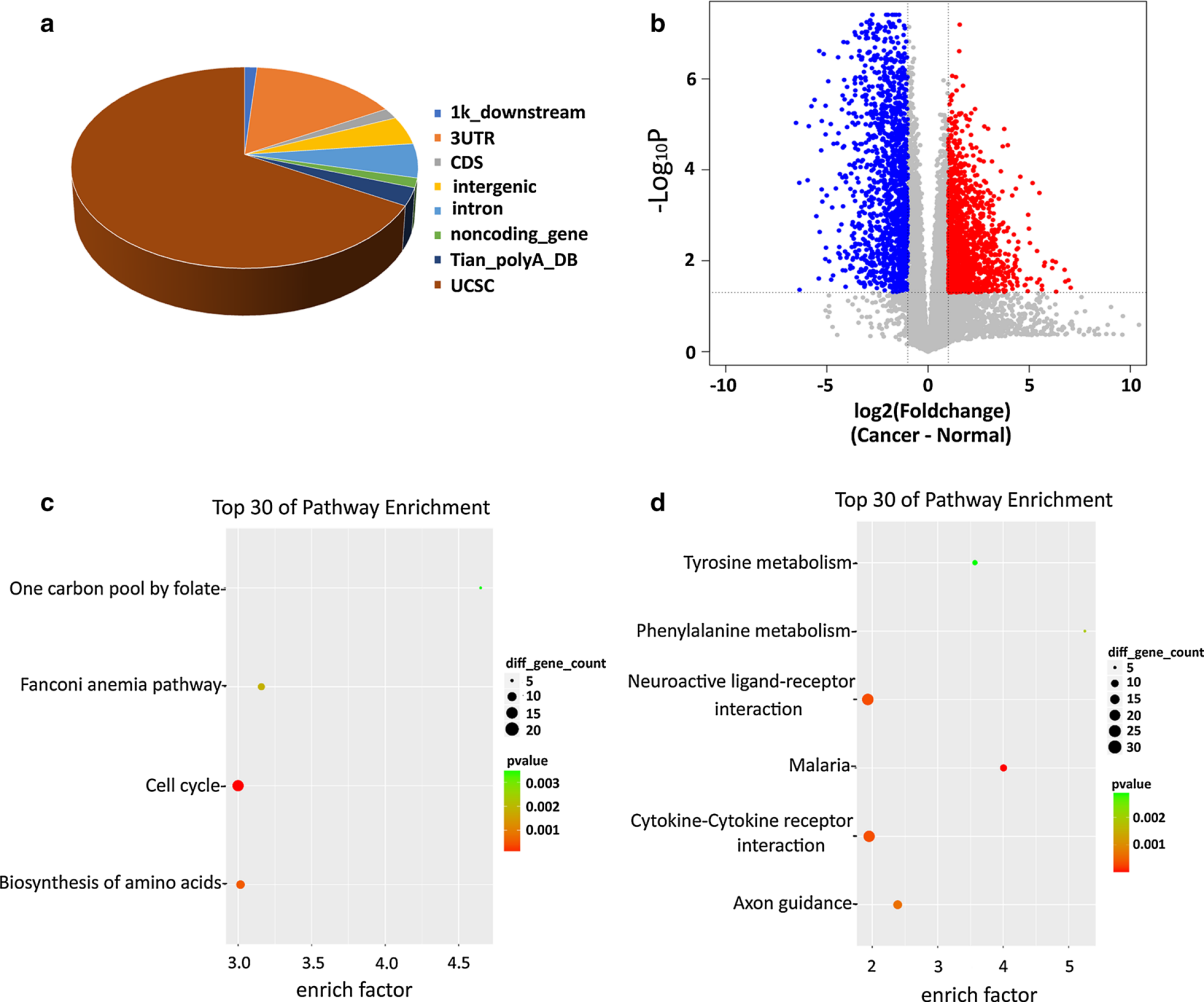
Characteristics of poly(A) sites. The reads and poly(A) sites were first mapped to the known sites of UCSC and Tiaqn’s poly(A) database, and the unmapped reads were annotated to 3′UTR, intron, CDS, 1 kb-downstream and intergenic region. **a** Pie-chart of mapping location of reads. **b** Volcano plot for DEGs. **c** KEGG enrichment analysis for up-regulated genes in cancer tissues. **d** KEGG enrichment analysis for down-regulated genes in cancer tissues

From a total of 41,773,101 average reads per each paired sample that were obtained from IVT-SAPAS analysis, we found an average read of 17,260,816 that meet the criteria for data analysis after data filtering, genome alignment and orientation filtering (Additional file 1: Table S1). Of them, 5,303,272 reads correspond to APA sites, with near 69.7% reads being mapped to the transcript of the UCSC ends database and Tian’s database, and 1.5% and 1.3% of the reads being mapped to the noncoding gene or 1 kb downstream of the UCSC canonical genes defined using the GENCODE gene loci, respectively (Fig. 1a). In addition, there were 5.5%, 15.6% and 1.7% of reads were mapped in the introns, 3′UTRs or coding sequences (CDSs), respectively.

### Differentially expressed genes (DEGs) in NSCLC

We conducted a gene expression survey for lung cancer tissue versus paired non-carcinoma tissue by calculating the number of transcript reads. Based on this analysis, we identified a total of 14,030 genes with distinct difference of expression. Of them, 1439 (10.26%) genes showed up-regulated expression and 1364 (9.72%) genes showed down-regulated expression in lung cancerous tissue (Fig. 1b). KEGG (Kyoto Encyclopedia of Genes and Genomes) enrichment analysis further revealed that the up-regulated genes in cancer tissues were enriched in signaling pathways primarily associated with cell cycle and biosynthesis of amino acids (Fig. 1c), and the down-regulated genes were enriched in pathways of neuroactive ligand-receptor interaction and cytokine–cytokine receptor interaction (Fig. 1d).

### Identification of APA switching genes in NSCLC

**Fig. 2:**
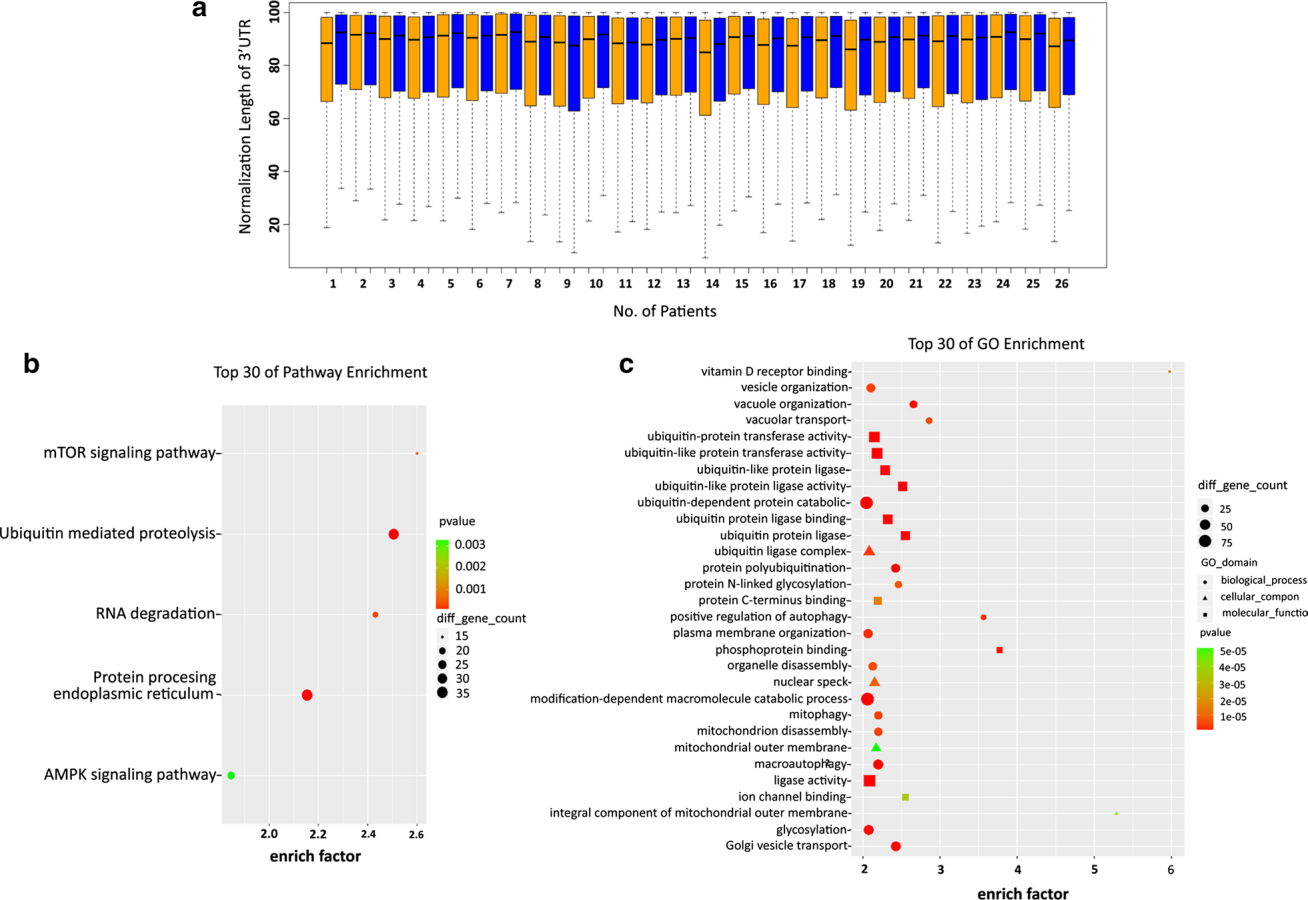
Differentially expressed genes in NSCLC tissue and paired para-carcinoma tissues and DAVID program-based functional annotation. **a** Comparison of the tandem 3′UTR standardized lengths of lung cancer tissue and paired para-carcinoma tissue from 26 pairs of clinical samples. Orange: cancer tissues. Blue: paired para-carcinoma tissues. **b** Enrichment of genes with significantly shorter 3′UTR isoforms involved in KEGG categories. **c** Enrichment of genes with significantly shorter 3′UTR isoforms involved in GO functional analysis

Previous studies have demonstrated that highly proliferative cells or cancer cells often expressed substantial amounts of mRNA isoforms with shortened 3′UTRs [6, 17]. Studies also revealed that shortened 3′UTRs correlated with poor prognosis of cancer, including lung cancer [13, 18]. We thus performed a comparison analysis for the tandem 3′UTRs lengths of these identified genes. For this, we set up a standard of the 3′UTR length by designating the longest 3′UTR as 1.0 and calculated the 3′UTR length of each gene using weighted mean of 3′UTR length with multiple APA sites to reduce the variance of 3′UTR length. We detected shortened 3′UTR in collected cancer tissues from 96.2% (25/26) patients, as shown in Fig. 2a. We also performed a test of linear trend to determine the tandem 3′UTR length switching. Based on the statistical significance (FDR = 0.01, |r| ≥ 0.1), we identified total of 7639 genes with potential APA switching in 26 paired clinical samples. Of interest, we noticed that lung cancer cells tend to have shortened 3′UTRs of these identified genes (1855 genes showing shorten 3′UTR and 58 genes showing elongated 3′UTR). Additional file 1: Table S2 shows the gene numbers with identified APA switching for each patient.

To determine the significance of these APA site-switching events, we performed functional annotation using the web-accessible DAVID program. The analysis indicated that genes with shorten 3′UTR are enriched in signaling pathways such as mTOR signaling, ubiquitin mediated proteolysis and RNA degradation (Fig. 2b). With gene ontology (GO) enrichment analysis, we also identified the top 30 enriched genes with shorten 3′UTR determined in these clinical samples (Fig. 2c).

### CSTF2 regulates 3′UTR shortening in NSCLC cell

**Fig. 3:**
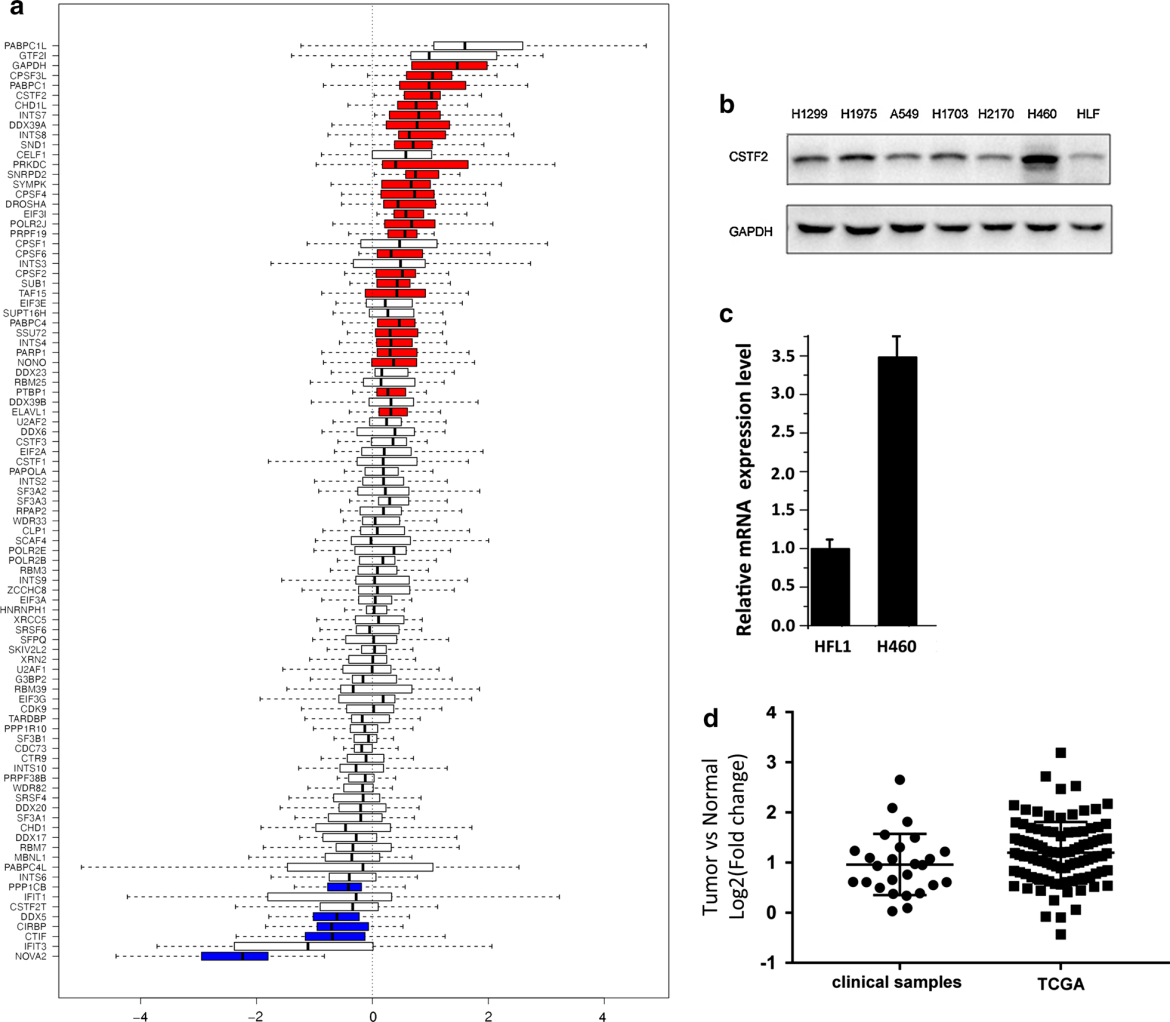
CSTF2 expression in lung cancer cells. **a** Log2 results for ratio of the gene expression level between cancer and para-carcinoma tissue of 93 RNA transcription and translation related influential factors. Red colored bars represented the genes with significantly up-regulated expression (p < 0.01) in cancer tissue. Blue colored bars represented the genes with significantly down-regulated expression (p < 0.01) in cancer tissue. **b** Western blot analysis showing the CSTF2 protein levels of lung cancer cell lines and normal lung HFL1 cell line. **c** Graph showing the higher mRNA expression of CSTF2 detected in the H460 compared to HFL1 cells. Data represents the average of three independent q-RT-PCR experiment. **d** Log2 results for ratio of CSTF2 gene expression detected in our clinical samples and summarized from TCGA database

To assess the potential mechanisms of 3′UTR shortening in human lung cancer, we examined the RNA expression of genes (Additional file 1: Table S3) that their encoding proteins may correlate to 3′UTR length editing. Of total 93 candidate genes, we found that 28 genes displayed significant changes of up-regulation and 5 genes of down-regulation. Our results also revealed that CSTF2 was one of the top genes with most significant up-regulation in cancerous tissues versus paired non-cancerous tissues (Fig. 3a and Additional file 1: Tables S3, S4). Of interest, we detected higher protein expression of CSTF2 in six NSCLC cancer cell lines versus normal lung HFL1 cell line. Of these established cancer cell lines, H460 cells showed highest expression level of CSTF2 (Fig. 3b). Our qRT-PCR analysis further confirmed the higher mRNA expression of CSTF2 in H460 cells when comparing to HFL1 cells (Fig. 3c). We further assess the expression pattern of CSTF2 in lung cancer samples in The Cancer Genome Atlas database (TCGA), and the results from 100 paired TCGA clinical samples showed near twofolds change of up-regulated CSTF2 expression in cancer cells versus normal lung cells, which is in consistence with the CSTF2 expression pattern of our clinical samples (Fig. 3d). We thus hypothesize that the CSTF2 gene may play a key role in regulation of 3′UTR length in lung cancer cells.

**Fig. 4:**
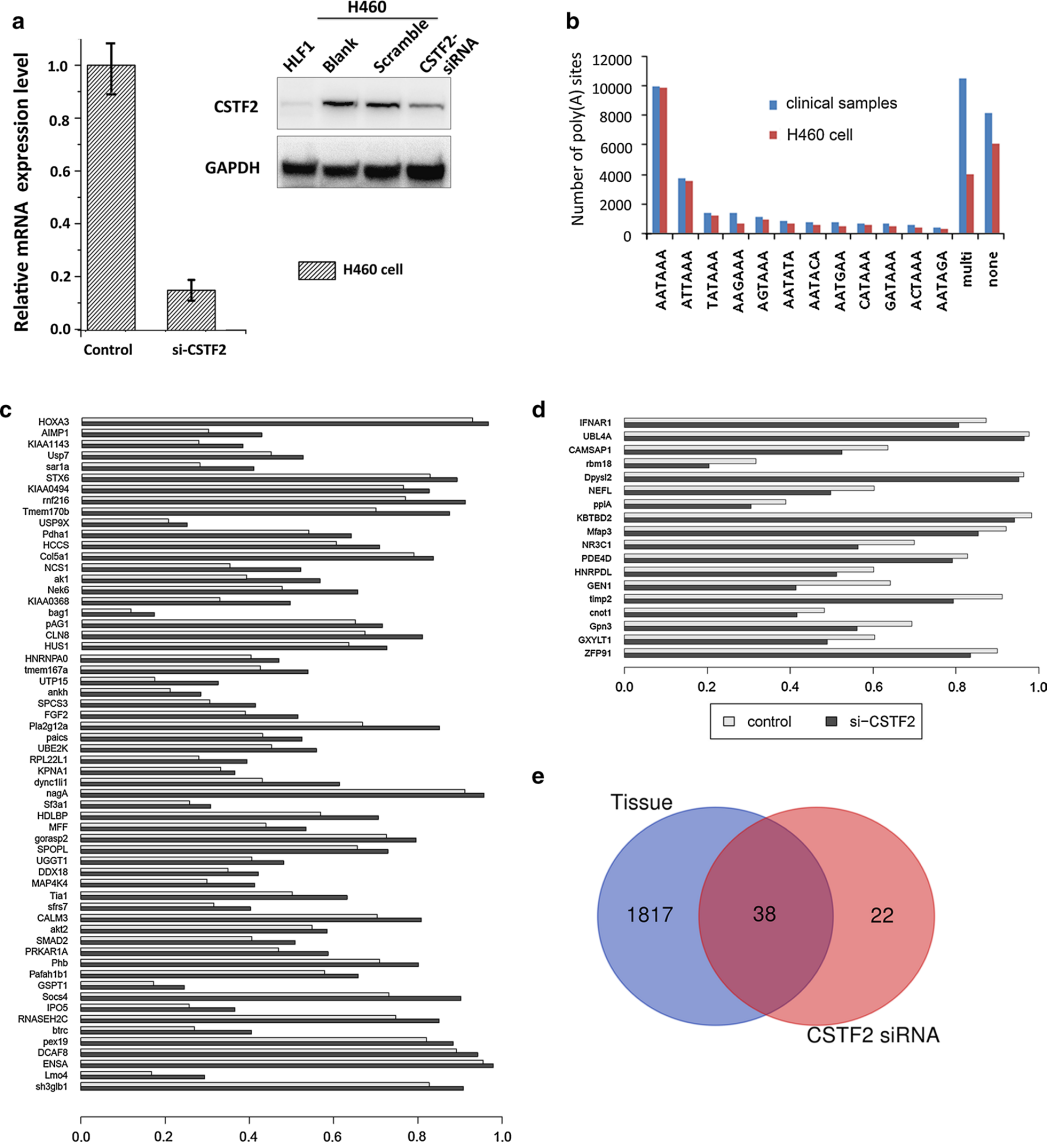
CSTF2 regulates 3′UTR shortening of genes in NSCLC cell. **a** siRNA transduction reduces the mRNA (left) and protein (right) levels of CSTF2 gene in H460 cells. **b** Comparison of distribution pattern for poly(A) signals in data sets of clinical samples and H460 cells. **c** Profiling of 60 genes with elongated 3′UTR length in H460 cells with knocking-down of CSTF2 expression. **d** Profiling of 18 genes with shortened 3′UTR length in H460 cells with knocking-down of CSTF2 expression. **e** Venn diagram for comparison between overlapped genes detected in data sets of H460 cells and clinical samples

To test this, we used RNA interference to knock down the CSTF2 expression in H460 cells, and determined the APA site-switching events with IVT-SAPAS analysis. We observed near 80% knocking-down effect of siRNA on CSTF2 expression (Fig. 4a), and a total of 10,235 genes with significant change after siRNA transduction was detected in H460 cells. Of them, 5694 genes (55.63%) are with APA site-switching events, 269 genes (2.62%) show expression difference, and 78 genes (0.76%) show distinct change of 3′UTR length. Of note, the global comparison shows consistence of IVT-SAPAS reads for genes with APA site switching between data sets for the clinical samples and for H460 cells, however, significant differences are observed for genes with differential expression or with different 3′UTR in length, whereas more reads were detected with clinical sample set (Additional file 1: Table 5S). In addition, we noticed that the IVT-SAPAS reads from the clinical samples and H460 cells showed similar distribution pattern of poly(A) signals (6-nucleotides), which is consistent with the previously reported [15] and demonstrates the validity of the IVT-SAPAS analysis used in our study capturing poly(A) site (Fig. 4b).

In IVT-SAPAS assay for H460 set, we detected elongation of 3′UTR length for 60 genes in CSTF2-siRNA transducted cells (Fig. 4c). Of interest, we found that, of the 1855 genes with shorter 3′UTR length identified in human lung cancer tissues (Fig. 4d), 38 genes displayed elongation of 3′UTR length when CSTF2 expression was knocked down in H460 cells (Fig. 4e and Table 2). The functional annotation of these genes further revealed that eleven of these 38 overlapping genes can be annotated into signaling pathways of insulin signaling, lipid metabolism and MAPK signaling. For example, FGF2 (fibroblast growth factor 2, uc003iev.1), which shows 0.3 fold-change of down-regulation in cancer tissue (P-value = 1.85e−4), has been demonstrated to play vital roles in tumor angiogenesis and lymphangiogenesis of lung cancer [19, 20]; MAP4K4 (mitogen-activated protein kinase kinase kinase kinase 4, uc002tbc.2), which shows 1.2 fold-change of upregulation in cancer tissue (P-value = 0.037), contributes to cell proliferation, anchorage-independent growth and cancer cell migration of lung cancer [21]. Of interest, the analysis for the distributions of poly(A) reads showed switching of distal stop codon in clinical samples may indicate cancer cells prefer to use the shorten length of transcripts for both FGF2 and MAP4K4 when comparing to normal cells, and the knocking down of CSTF2 expression in H460 cells could lead to changes of transcript lengths of these genes according to the detected numbers and sites of poly(A) reads (Additional file 2: Fig. S1). These findings thus not only demonstrate the vital role of CSTF2 in regulation of 3′UTR length for cancer cell-associated genes, but also indicate a potential of CSTF2 or its protein family may also serve as oncogene(s) driving carcinogenesis of NSCLC.

**Table 2:** KEGG pathway analysis of 38 overlapping genes in si-CSTF2 cell line

UCSC ID	Gene name	KEGG_PATHWAY
uc001dlw.2	SH3-domain GRB2-like endophilin B1	hsa04144:Endocytosis
uc001kta.2	Beta-transducin repeat containing	hsa04114:Oocyte meiosis, hsa04120:Ubiquitin mediated proteolysis, hsa04310:Wnt signaling pathway, hsa04340:Hedgehog signaling pathway
uc002pew.2	Calmodulin 3 (phosphorylase kinase, delta); calmodulin 2 (phosphorylase kinase, delta); calmodulin 1 (phosphorylase kinase, delta)	hsa04020:Calcium signaling pathway, hsa04070:Phosphatidylinositol signaling system, hsa04114:Oocyte meiosis, hsa04270:Vascular smooth muscle contraction, hsa04720:Long-term potentiation, hsa04722:Neurotrophin signaling pathway, hsa04740:Olfactory transduction, hsa04910:Insulin signaling pathway, hsa04912:GnRH signaling pathway, hsa04916:Melanogenesis, hsa05010:Alzheimer’s disease, hsa05214:Glioma
uc003iev.1	Fibroblast growth factor 2 (basic)	hsa04010:MAPK signaling pathway, hsa04810:Regulation of actin cytoskeleton, hsa05200:Pathways in cancer, hsa05218:Melanoma
uc002tbc.2	Mitogen-activated protein kinase kinase kinase kinase 4	hsa04010:MAPK signaling pathway
uc003hzp.2	Phospholipase A2, group XIIA	hsa00564:Glycerophospholipid metabolism, hsa00565:Ether lipid metabolism, hsa00590:Arachidonic acid metabolism, hsa00591:Linoleic acid metabolism, hsa00592:alpha-Linolenic acid metabolism, hsa04010:MAPK signaling pathway, hsa04270:Vascular smooth muscle contraction, hsa04370:VEGF signaling pathway, hsa04664:Fc epsilon RI signaling pathway, hsa04730:Long-term depression, hsa04912:GnRH signaling pathway
uc003hbs.1	Phosphoribosylaminoimidazole carboxylase, phosphoribosylaminoimidazole succinocarboxamide synthetase	hsa00230:Purine metabolism
uc002fuw.3	Platelet-activating factor acetylhydrolase, isoform Ib, subunit 1 (45 kDa)	hsa00565:Ether lipid metabolism
uc002jhg.2	Protein kinase, cAMP-dependent, regulatory, type I, alpha (tissue specific extinguisher 1)	hsa04210:Apoptosis, hsa04910:Insulin signaling pathway,
uc004czg.3	Pyruvate dehydrogenase (lipoamide) alpha 1	hsa00010:Glycolysis/Gluconeogenesis, hsa00020:Citrate cycle (TCA cycle), hsa00290:Valine, leucine and isoleucine biosynthesis, hsa00620:Pyruvate metabolism, hsa00650:Butanoate metabolism,
uc001xbo.2	Suppressor of cytokine signaling 4	hsa04630:Jak-STAT signaling pathway, hsa04910:Insulin signaling pathway, hsa04930:Type II diabetes mellitus

## Discussion

In eukaryotic cells, polyadenylation, a process of adding poly(A) tails to of nearly every fully processed mRNA, is the last key step in mRNA maturation and has been suggested to play critical roles in virtually all aspects of a transcript’s life cycle such as mRNA synthesis, conferring mRNA stability, mRNA transportation from nucleus to the cytoplasm and promoting mRNA’s translational efficiency [22–26]. The use of tandem APA sites located on the terminal exons often leads to tandem 3′UTRs with variable lengths, and this 3′UTR-APA event plays important roles in regulating the gene expression network and is globally regulated in response to changes in cell proliferation and differentiation [11].

Global 3′UTR regulation corresponding to the changes of cellular proliferation and differentiation status has been observed in cancer cells. Mayr et al. reported the results of using the 3′RACE assay for the change of APA on oncogene activation [6]. They found the genes with alternative isoforms can be detected in all the tested cancer cell lines (Cyclin D1, DICER1 and RAB10), or in at least a third of the samples (IMP-1, Cyclin D2 and FGF2), and shorter mRNAs were detectable and more prominent in these cancer cell lines compared to normal tissues. In this study, we found that 3′UTR shortening is a common event for cancer-related genes and shorter mRNAs are more frequently detected in tumor tissues compared to corresponding normal tissues. Of note, of the identified 1855 genes (FDR = 0.01) with a significant difference of the 3′UTR length that may link tandem 3′UTR length switching with the pathologic process of lung cancer, we detected alternative isoforms of Cyclin D1, DICER1, RAB10, Cyclin D2, FGF2 in all clinical cancer samples, and alternative isoform of IMP-1 in a remarkable portion of the samples (Additional file 3: Fig. S2). Consistently with Mayr’s study, these genes expressed a higher fraction of shorter APA isoforms in a majority of the cancer tissues comparing to the corresponding normal tissues (Additional file 1: Table S6). In addition to these findings, our results further revealed that these identified genes with shorten 3′UTR were enriched in signaling pathways of mTOR signaling, ubiquitin mediated proteolysis and RNA degradation. These results provide novel insights for the contribution of 3′UTR-APA site-switching events of cancer-related genes to cancer development and for development of novel therapeutic strategies of lung cancer.

We also performed the histological comparison of IVT-SAPAS reads for adenocarcinoma and squamous lung cancer with our data set of the clinical samples. Our results showed that 719 down-regulated and 1045 up-regulated genes were detected in both histological types of cancers. Of interest, 16 genes with APA shortness and only one gene with APA lengthen were detected in both adenocarcinoma and squamous lung cancer tissues (Additional file 4: Fig. S3 and Additional file 1: Table S7). These overlapped gene events may indicate the privilege of certain genes or profile of genes to be involved in the cancer development and disease progression regardless of the histological difference for NSCLC. It is needed to be indicated that, however, the initial results from the IVT-SAPAS data set with clinical samples showing the histological similarity of genes with APA switching for adenocarcinoma and squamous lung cancer needs to be further identified due to the limited number of clinical samples enrolled.

Our data also revealed the potential roles of polyadenylation processing protein, CSTF2, on the shortness of 3′UTR of these cancer-related genes. Processing at the 3′-end is directed by sequence elements in the pre-mRNA (cis elements) and requires a large number of protein factors that functionally and physically interacts each other between the termini of an mRNA. Currently, more than fourteen proteins have been identified in four multi-subunit protein complexes of human polyadenylation machinery for mRNA 3′-end processing, including binding of poly(A) binding protein (PABP), cleavage and polyadenylation specificity factor (CPSF), cleavage stimulation factor (CSTF), cleavage factors I (CF Im) and cleavage factors II (CF IIm) [27, 28]. CSTF is essential for the cleavage reaction [28, 29]. CSTF complex is composed of three subunits of molecular weights 50 (CSTF1), 64 (CSTF2), and 77 (CSTF13) kDa [30]. CSTF-64 (also known as CSTF2) was one of the first proteins identified, based on its strong and specific UV cross-linking to RNAs containing a functional poly(A) signal [31], within the 3′-end processing complex of pre-mRNA. CSTF-64 contains a conserved RNA recognition motif (RRM) RNA binding domain at its N-terminus, with the presence of a UU dinucleotide-specific binding site and highly mobilized protein: RNA interface which allows CstF-64 to form stable complexes with a wide range of GU-rich sequences [27, 31, 32]. Recent study has demonstrated that CstF64 is also important as a regulator for APA [33]. Our data present in this study showed that the expression of CSTF2 correlates to the shortening of 3′UTR of the differentially expressed genes in lung cancer, and the results from this study suggest a clinic potential of CSTF2 as a therapeutic target for lung cancer.

## Conclusion

In summary, our data present here demonstrated that APA site-switching of 3′UTRs is prevalent in NSCLC, and APA-mediated regulation of gene expression may play important roles in development and progression of NSCLC. Our results also revealed that regulatory role of CSTF2, a polyadenylation processing protein, on the shortening of 3′UTR of these cancer-related genes, and a potential of CSTF2 as a therapeutic target for lung cancer.

## Additional files

**Additional file 1.** Additional tables.

**Additional file 2: Figure S1.** Comparisons of changes for FGF2 and MAP4K4 poly. (A) Expression of poly(A) isoform in data sets of clinical samples and H460 cell line. Lung tumor cells prefers to use the shorten length transcripts of FGF2 (A) and MAP4K4 (B) compared to para-cancer tissue cells, and knocking-down of CSTF2 expression in H460 cells leads to extended length of transcripts for both FGF2 and MAP4K4 genes.

**Additional file 3: Figure S2.** Expressions of the gene with alternative isoforms. The y-axis indicated the APA site of each gene, the x-axis indicated the 26 cancer tissues. The scale bar indicated the expression level of each isoform.

**Additional file 4: Figure S3.** Histological comparison of IVT-SAPAS reads for clinical adenocarcinoma and squamous lung cancer samples in sets of down-regulated genes (top left), up-regulated genes (top right), APA shortened genes (bottom left) and APA lengthened genes (bottom right).

## Data Availability

The datasets used and/or analyzed during the current study are available from the corresponding author on reasonable request.

## Abbreviations

SCLC: small cell lung carcinoma
NSCLC: non-small cell lung carcinoma
3′-UTRs: 3′ untranslated regions
APA: alternative polyadenylation
CSTF: cleavage stimulation factor
RT: reverse transcription
DAVID: the database for annotation, visualization and integrated discovery
DEGs: differentially expressed genes
KEGG: Kyoto Encyclopedia of Genes and Genomes
UTR: untranslated regions
GO: gene ontology
PABP: poly(A) binding protein
CPSF: cleavage and polyadenylation specificity factor
RRM: RNA recognition motif
