# Supplementary figures and images for: Genome-wide profiling reveals alternative polyadenylation of mRNA in human non-small cell lung cancer
Source: J Transl Med. 2019 Aug 7;17:257. doi: 10.1186/s12967-019-1986-0 (PMC6686416; doi:10.1186/s12967-019-1986-0)

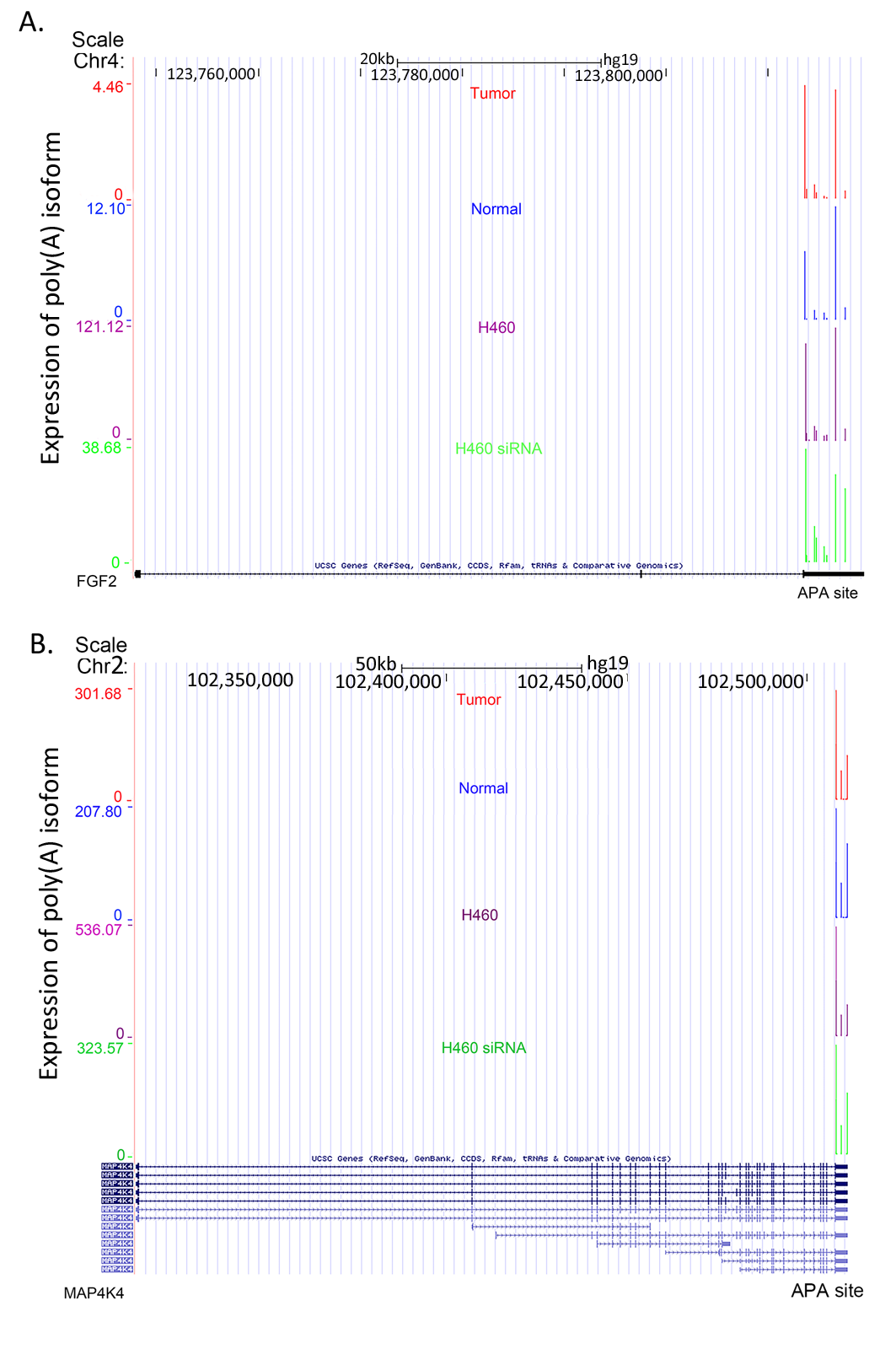

Supplement: Supplementary file 2 — Additional file 2: Figure S1. Comparisons of changes for FGF2 and MAP4K4 poly. (A) Expression of poly(A) isoform in data sets of clinical samples and H460 cell line. Lung tumor cells prefers to use the shorten length transcripts of FGF2 (A) and MAP4K4 (B) compared to para-cancer tissue cells, and knocking-down of CSTF2 expression in H460 cells leads to extended length of transcripts for both FGF2 and MAP4K4 genes. [file 12967_2019_1986_MOESM2_ESM.tif]

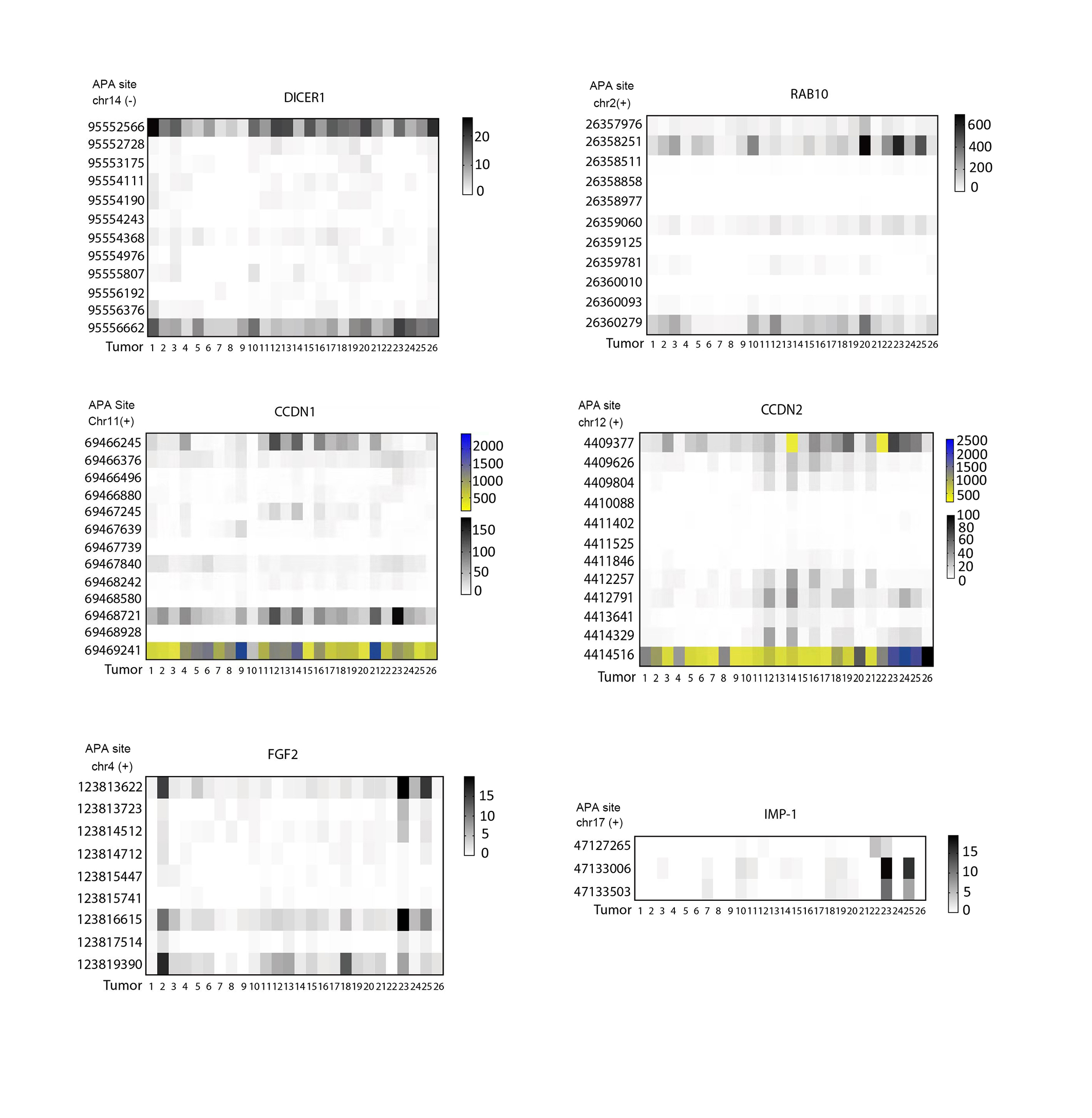

Supplement: Supplementary file 3 — Additional file 3: Figure S2. Expressions of the gene with alternative isoforms. The y-axis indicated the APA site of each gene, the x-axis indicated the 26 cancer tissues. The scale bar indicated the expression level of each isoform. [file 12967_2019_1986_MOESM3_ESM.tif]

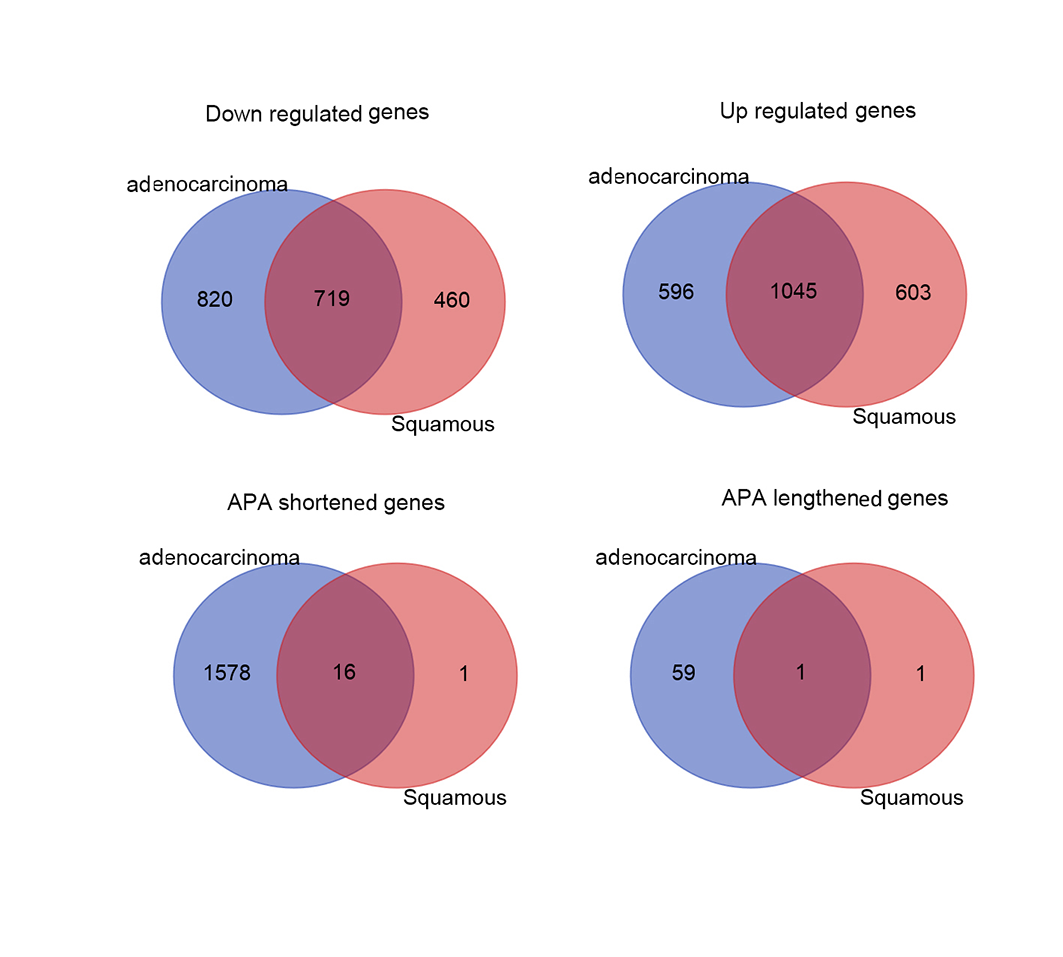

Supplement: Supplementary file 4 — Additional file 4: Figure S3. Histological comparison of IVT-SAPAS reads for clinical adenocarcinoma and squamous lung cancer samples in sets of down-regulated genes (top left), up-regulated genes (top right), APA shortened genes (bottom left) and APA lengthened genes (bottom right). [file 12967_2019_1986_MOESM4_ESM.tif]
